# Low Rates of Psychosocial Screening and Lifestyle Counseling in Hidradenitis Suppurativa Patients in the USA

**DOI:** 10.1159/000528253

**Published:** 2023-01-05

**Authors:** Terri Shih, Devea R. De, Jonathan Rick, Vivian Y. Shi, Jennifer L. Hsiao

**Affiliations:** ^a^David Geffen School of Medicine, University of California Los Angeles, Los Angeles, California, USA; ^b^University at Buffalo, Jacobs School of Medicine and Biomedical Sciences, Buffalo, New York, USA; ^c^Department of Dermatology, University of Arkansas for Medical Sciences, Little Rock, Arkansas, USA; ^d^Department of Dermatology, University of Southern California, Los Angeles, California, USA

**Keywords:** Hidradenitis suppurativa, Psychosocial screening, Lifestyle counseling

## Abstract

**Introduction:**

Although hidradenitis suppurativa (HS) is associated with psychosocial comorbidities such as depression as well as modifiable comorbidities such as obesity, rates of psychosocial screening and lifestyle counseling in the USA have not been characterized.

**Methods:**

This cross-sectional study utilized publicly available data from the National Ambulatory Medical Care Survey (NAMCS) between 2008 and 2018 to identify visits with a diagnosis of HS (ICD-9 code 705.83, ICD-10 code L73.2). *T* tests and multivariate logistic regressions analyzed trends in rates of screening and counseling while controlling for race, sex, and age. Survey weights are applied to each visit to represent a national sample.

**Results:**

Depression screening was completed in only 2% of reported visits. No visits reported screening for alcohol misuse, substance abuse, or domestic violence. There were low rates of counseling for weight reduction (7.8%), diet and nutrition (3.3%), exercise (2.4%), smoking (1.0%), and substance abuse (0.7%). Black patients and individuals with public health insurance received less screening and counseling overall.

**Conclusion:**

Rates of psychosocial screening and counseling on lifestyle modifications are low in ambulatory clinic visits for HS patients, and there are disparities based on race and insurance status. Implementing strategies to incorporate routine psychosocial screening and lifestyle counseling into visits may improve HS patient outcomes.

## Introduction

Hidradenitis suppurativa (HS) is a chronic, debilitating inflammatory skin condition characterized by painful nodules, abscesses, sinus tracts, and scarring that imparts significant physical and psychosocial burdens. Associated comorbidities include smoking, obesity, metabolic syndrome, cardiovascular disease, depression, anxiety, and substance use disorder, among others [1]. Risk of intimate partner violence has been reported to be 2.4 times more likely in individuals with HS as compared to those with acne [2]. Screening for psychosocial conditions such as depression and domestic abuse and counseling for lifestyle modifications such as diet and exercise are important components of a comprehensive care strategy for HS. However, few studies have characterized how frequently this screening or counseling occurs for patients with HS. Herein, we examine characteristics of HS ambulatory visits and the rates of psychosocial screening and counseling in patients with HS in the USA.

## Methods

The National Ambulatory Medical Care Survey (NAMCS) is conducted annually by the National Center for Health Statistics from the Centers for Disease Control and Prevention, which utilizes a stratified, random sample of patient visits to nonfederal, ambulatory office-based physicians. Physicians are randomly assigned a 1-week reporting period. A random sample of visits is assessed for data on patient demographics and symptoms and physician diagnoses and management, including screening and counseling, medications prescribed, and procedures completed. Survey weights are applied to each visit to represent a national sample.

In this study, we searched publicly available NAMCS data between 2008 and 2018 (2017 was unavailable) for visits with a diagnosis of HS (ICD-9 code 705.83, ICD-10 code L73.2). Descriptive statistics were completed for demographic data and rates of psychosocial screening and lifestyle modification counseling. *T* tests and multivariate logistic regressions analyzed trends in rates of screening and counseling while controlling for race, sex, and age. Multivariate race comparisons excluded the category of race reported as “other” due to small sample size. Visits with missing data in relevant analyses were excluded. All data analyses were performed using SAS Studio 9.04.01 (SAS Institute, Cary, NC, USA). Variance in the complex survey design is accounted for by utilizing survey weights to create national estimates and confidence intervals (CI).

## Results

From the 2008–2018 NAMCS datasets, an estimated 2.33 million visits (95% CI, 1.95 million–2.71 million) had a diagnosis of HS. Of these, 71.1% of the patients were female, 75.6% were white, and the mean age was 37.9 ± 1.0 (range 12–69) (Table 1).

Depression screening was completed in a small minority (2.0%) of visits, none of which were completed in black patients (Table 2). Depression screenings were slightly less likely to be conducted in older patients (OR, 0.94 [95% CI, 0.91–0.98], *p* = 0.003). No visits reported screening for alcohol misuse, substance abuse, or domestic violence.

Physicians reported overall low rates of counseling for weight reduction (7.8%), diet and nutrition (3.3%), exercise (2.4%), smoking (1.0%), and substance abuse (0.7%) (Table 2). Black patients were more likely to be counseled on weight reduction (OR, 4.95 [95% CI, 2.02–12.13], *p* = 0.003) but less likely to receive diet and nutrition counseling (OR, 0.52 [95% CI, 0.32–0.85], *p* = 0.01). Of visits that reported counseling on exercise, substance abuse, and tobacco use, none were completed in black patients. Older patients were slightly less likely to receive counseling on diet/nutrition (OR, 0.98 [95% CI, 0.96–1.00], *p* = 0.01), exercise (OR, 0.94 [95% CI, 0.91–0.97], *p* = 0.001), and weight reduction (OR, 0.90 [95% CI, 0.87–0.94], *p* < 0.001). There was no statistically significant difference in rates of counseling between men and women. Patients with higher BMI were more likely to receive counseling on exercise (OR, 1.24 [95% CI, 1.09–1.41], *p* = 0.002), weight reduction (OR, 1.09 [95% CI, 1.06–1.12], *p* < 0.001), and diet/nutrition (OR, 1.07 [95% CI, 1.05–1.08], *p* < 0.001) after controlling for age, sex, and race.

Visits funded by public insurance including Medicare and Medicaid less frequently received counseling overall. They were significantly less likely to receive counseling on weight reduction (OR, 0.08 [95% CI, 0.01–0.79], *p* = 0.03).

## Discussion

Rates of psychosocial screening and lifestyle counseling at ambulatory visits were low among patients with HS. Overall, individuals who were black or had public health insurance received less depression screening and lifestyle counseling.

Given rates of depression in HS patients have been found to be as high as 26% and there is an increased risk of suicide in HS patients [3], routine depression screening is warranted [1]. However, the rate of depression screening was found to be only 2% in ambulatory clinic visits for HS patients in our study. The rate of substance use disorder in the USA has been found to be 4% in HS patients versus 2% in control patients [4]. One cross-sectional study interviewed 243 Canadian patients (128 with HS, 115 with acne) and found 2.4 times of risk of intimate partner violence compared to patients with acne [2]. However, none of the visits across the 10-year span of our study reported screenings on substance use and domestic violence, highlighting a potential practice gap.

HS is associated with smoking, obesity, and poor cardiovascular outcomes [1, 5]. Though more data are needed, studies have suggested a correlation between smoking status and HS severity and duration [6, 7], and weight reduction has been linked to HS disease improvement [8]. Regardless of impact on HS disease activity, counseling on lifestyle modifications for diet, exercise, and smoking cessation should be performed for the overall health of HS patients. Of note, addressing lifestyle changes after the first establishing rapport with patients is helpful [9].

Racial and socioeconomic disparities were observed in the rates of depression screening and lifestyle counseling in patients with HS. Black patients and individuals with public health insurance received less screening and lifestyle counseling overall. It is imperative that depression screening and lifestyle counseling increase for all patients with HS, with particular attention paid to underserved populations. This is especially noteworthy as black patients and patients with low socioeconomic status are disproportionately affected with HS [10, 11].

Limitations of the NAMCS database include lack of data on HS severity. Given HS is associated with delayed and missed diagnoses [12], the number of HS ambulatory visits may be underrepresented. The NAMCS database may not capture all performed screenings for depression and other psychosocial conditions or counseling of lifestyle modifications. Given overall low-estimated total HS visits and screening and counseling rates, comparisons were not made across provider specialties. In addition, visits with missing data were excluded in our analyses.

Underscreening for depression and substance abuse in HS patients may be due to lack of awareness. Additionally, integrating mental health screening and lifestyle modification counseling into time-constrained clinic visits may be challenging. Quick screening measures such as the Patient Health Questionnaire-2 for depression and implementation of streamlined mental health referral pathways may be useful [13]. Providing handouts on lifestyle modifications can increase patient's understanding of their comprehensive management plan in an efficient manner [14]. All specialties caring for HS patients should aim to incorporate psychosocial screening and lifestyle counseling into their care to improve patient outcomes.

## Statement of Ethics

Ethical approval and consent were not required as this study was based on publicly available data. The National Center for Health Statistics (NCHS) Ethics Review Board reviews the content of the National Ambulatory Medical Care Surveys to ensure compliance with NCHS practices and procedures. Additional information can be found on www.cdc.gov/nchs/ahcd/index.htm. The National Ambulatory Medical Care Surveys fall under Title 42, US Code, section 242K, which permits data collection for health research. NCHS will not disclose responses in identifiable form without the consent of the individual or establishment in accordance with section 308(d) of the Public Health Service Act and the Confidential Information Protection and Statistical Efficiency Act of 2018. Additional information can be found on www.cdc.gov/nchs/ahcd/index.htm.

## Conflict of Interest Statement

Jennifer L. Hsiao is on the board of directors for the Hidradenitis Suppurativa Foundation; has served as a consultant for Boehringer Ingelheim, Novartis, and UCB; and has served as a consultant and speaker for AbbVie. Vivian Y. Shi is on the board of directors for the Hidradenitis Suppurativa Foundation (HSF); is a stock shareholder of Learn Health; and has served as an advisory board member, investigator, speaker, and/or received research funding from Sanofi Genzyme, Regeneron, AbbVie, Eli Lilly, Novartis, SUN Pharma, LEO Pharma, Pfizer, Incyte, Boehringer Ingelheim, Aristea Therapeutics, Menlo Therapeutics, Dermira, Burt's Bees, Galderma, Kiniksa, UCB, WebMD, TARGET Pharmasolutions, Altus Lab, MYOR, Polyfin, GpSkin, and Skin Actives Scientific. There was no financial transaction for the preparation of this manuscript. All other authors report no conflicts of interest.

## Funding Sources

This article has no funding source.

## Author Contributions

Terri Shih and Jonathan Rick completed data analysis. Terri Shih and Devea R. De drafted the manuscript. Jonathan Rick, Vivian Shi, and Jennifer Hsiao edited and reviewed the manuscript. Vivian Shi and Jennifer Hsiao conceptualized and led the project.

## Data Availability Statement

All data files are available from publicly available websites accessible through the CDC website, www.cdc.gov/nchs/ahcd/index.htm. Further inquiries can be directed to the corresponding author.

## Figures and Tables

**Table 1:** Survey-weighted visit demographics and characteristics of HS visits

Demographics	% of total	95% CI
Age, years, mean±SD (range)	37.9±1.0 (12–69)	
0–17	3.7	0.0–7.5
18–39	54.7	45.2–64.2
40–59	31.5	20.8–42.3
60+	10.1	6.3–13.9
Female	71.1	59.6–82.6
Hispanic or Latino	5.7	0.4–11.0
Race		
White	75.6	67.9–83.3
Black	23.0	15.4–30.6
Other	1.4	0.6–2.2
Types of payment		
Private insurance	57.2	45.6–68.7
Non-Medicare public health insurance program	29.7	18.6–40.7
Medicare	8.8	5.0–12.6
Self-pay	1.1	0.0–2.6
Specialty of visit provider		
Dermatology	30.2	20.5–39.9
Family practice	24.2	16.4–32.0
General surgery	18.9	13.2–24.6
Internal medicine	6.6	0.0–15.8
Pediatrics	1.2	0.1–2.4
Obstetrics and gynecology	0.4	0.3–0.4

**Table 2:** Multivariate comparisons of screening and counseling rates during survey-weighted ambulatory visits*

	Overall	Sex			Race			Insurance status^a^		
		female	male	*p* value	white	black	*p* value	Public	other	*p* value
Depression screening^b^	2.0 (1.3–2.7)	1.7 (1.4–2.0)	0.3 (0.0–1.0)	048	2.0 (1.3–2.8)	0	−	0.3 (0.0–1.0)	1.7 (1.4–2.0)	0.45
Weight reduction counseling	7.8 (6.4–9.2)	7.1 (5.9–8.2)	0.7 (0.1–1.4)	0.72	3.1 (2.3–3.9)	4.8 (4.0–5.6)	0.001	0.3 (0.0–1.0)	7.5 (6.3–8.7)	0.03
Diet/nutrition counseling	3.3 (2.1–4.6)	2.2 (1.8–2.5)	1.2 (0.0–2.3)	0.58	2.9 (1.7–4.1)	0.5 (0.4–0.5)	0.01	0.8 (0.1–1.4)	2.5 (1.5–3.5)	0.27
Exercise counseling	2.4 (1.7–3.2)	1.7 (1.4–2.0)	0.7 (0.1–1.4)	0.94	2.5 (1.7–3.2)	0	−	0.3 (0.0–1.0)	2.1 (1.8–2.5)	0.33
Tobacco use/exposure counseling	1.0 (0.3–1.6)	0.6 (0.5–0.7)	0.3 (0.0–1.0)	0.97	1.0 (0.3–1.6)	0	−	0.3 (0.0–1.0)	0.6 (0.5–0.7)	0.78
Substance abuse counseling	0.7 (0.0–2.1)	0	0.7 (0.0–2.1)	−	0.7 (0.0–2.2)	0	−	0.7 (0.0–2.1)	0	−

a Public insurance includes Medicare, Medicaid, Children's Health Insurance Program, and other state-based programs. “Other” includes all other types of payment method.

b No visits reported screening for alcohol misuse, substance abuse, or domestic violence.

* Data are presented as percentage of total visits (95% confidence interval).
